# High SIRT1 expression is a negative prognosticator in pancreatic ductal adenocarcinoma

**DOI:** 10.1186/1471-2407-13-450

**Published:** 2013-10-02

**Authors:** Albrecht Stenzinger, Volker Endris, Frederick Klauschen, Bruno Sinn, Katja Lorenz, Arne Warth, Benjamin Goeppert, Volker Ehemann, Alexander Muckenhuber, Carsten Kamphues, Marcus Bahra, Peter Neuhaus, Wilko Weichert

**Affiliations:** 1 Institute of Pathology, and National Center for Tumor Diseases (NCT), University Hospital Heidelberg, Heidelberg, Germany; 2 Institute of Pathology, Charité-Universitätsmedizin, Berlin, Germany; 3 Department of General, Visceral and Transplantation Surgery, Charité-Universitätsmedizin, Berlin, Germany

**Keywords:** Pancreatic cancer, HDAC, Sirt1, Biomarker, Pancreatic ductal adenocarcinoma

## Abstract

**Background:**

Several lines of evidence indicate that Sirt1, a class III histone deacetylase (HDAC) is implicated in the initiation and progression of malignancies and thus gained attraction as druggable target. Since data on the role of Sirt1 in pancreatic ductal adenocarcinoma (PDAC) are sparse, we investigated the expression profile and prognostic significance of Sirt1 *in vivo* as well as cellular effects of Sirt1 inhibition *in vitro*.

**Methods:**

Sirt1 expression was analyzed by immunohistochemistry in a large cohort of PDACs and correlated with clinicopathological and survival data. Furthermore, we investigated the impact of overexpression and small molecule inhibition on Sirt1 in pancreatic cancer cell culture models including combinatorial treatment with chemotherapy and EGFR-inhibition. Cellular events were measured quantitatively in real time and corroborated by conventional readouts including FACS analysis and MTT assays.

**Results:**

We detected nuclear Sirt1 expression in 36 (27.9%) of 129 PDACs. SIRT1 expression was significantly higher in poorly differentiated carcinomas. Strong SIRT1 expression was a significant predictor of poor survival both in univariate (p = 0.002) and multivariate (HR 1.65, p = 0.045) analysis. Accordingly, overexpression of Sirt1 led to increased cell viability, while small molecule inhibition led to a growth arrest in pancreatic cancer cells and impaired cell survival. This effect was even more pronounced in combinatorial regimens with gefitinib, but not in combination with gemcitabine.

**Conclusions:**

Sirt1 is an independent prognosticator in PDACs and plays an important role in pancreatic cancer cell growth, which can be levered out by small molecule inhibition. Our data warrant further studies on SIRT1 as a novel chemotherapeutic target in PDAC.

## Background

Pancreatic ductal adenocarcinoma (PDAC) is the fourth leading cause of cancer related deaths in the United States. While substantial progress has been made in the understanding of pancreatic cancer biology [1], therapeutic concepts still provide only modest benefit [2]. The overall 5-year survival rate is approximately 5% [3]. Surgical resection is the only efficient and potentially curative treatment option with 5-year survival rates of around 20% in patients with resectable tumors, but can only be applied in approximately 15-20% of the cases emphasizing the urgent need for early detection strategies [4].

The main prognosticators for surgically resectable PDACs are location, tumor size, resection margin, nodal status, and histological grade. Although these risk factors have been proven to be clinically useful, their ability to reliably predict outcome is limited and mainly reflects tumor distribution rather than tumor biology [4].

Hence, numerous studies have been conducted to identify novel biomarkers that aid outcome prediction and to unravel molecular mechanisms that drive tumor development [5].

Sirt1 (homolog of yeast silent information regulator, Sir2), an isoform of enzymes of the silent information regulatory family (sirtuins), is an evolutionary conserved NAD dependent histone/protein deacetylase (class III HDAC) that mediates epigenetic silencing by modification of lysine residues of histones and deacetylation of numerous non-histone substrates. One of the first substrates identified was p53, whose deacetylation was reported to repress p53-dependent apoptosis in response to cellular stress and DNA damage [6,7]. Meanwhile, many other targets have been identified, including Ku70 [8], PTEN [9], p73 [10], RelA/p65 [11], FOX01, FOX03a, and FOX04 [12], NICD [13], hypoxia-inducible factors HIF-1α, -2α [14,15], β-catenin [16], XPA [17], SMAD7 [18], and cortactin [19]. Deacetylation of these targets regulates cell survival, proliferation, and angiogenesis. Overexpression of sirtuins was initially reported to increase lifespan in budding yeast, Caenorhabditis elegans, and Drosophila melanogaster [20-22] but for the latter two the findings were challenged by a recent study of Burnett and colleagues [23].

The functional role of Sirt1 in cancer is equivocal and suggested to be context dependent [24]. Although there are convincing studies that argue for a tumour suppressive role of Sirt1, recent data provide functional evidence that Sirt1 has oncogenic properties in neuroblastomas by facilitating n-myc stabilization [25]. Serrano [26] reported that transgenic Sirt mice crossed with PTEN-null mice were observed to develop thyroid and prostate cancer further arguing for a tumor promoting function of Sirt1.

While several studies found deregulation of Sirt1 in various tumor entitites including ovary, prostate, gastric, colon, hepatocellular carcinoma as well as melanoma and glioblastoma [27], comprehensive *in vivo* data in pancreatic cancer is still missing. Reports that explore Sirt1 function in pancreatic cancer are sparse [28].

Hence, we set out to comprehensively investigate Sirt1 expression in a large series of PDACs, its relationship to survival and to assess the functional relevance in cell culture models.

## Methods

### Patients and samples

Tissue samples from 129 patients who underwent partial pancreaticoduodenectomy for primary pancreatic ductal adenocarcinoma between 1991 and 2000 were retrieved from the database of the Pathology Department of the Charité University Hospital. The study was approved by the Charité University Ethics Committee (No. EA1/06/2004).

Median age of patients with pancreatic cancer was 65 years (range 35–80 years). Follow-up data regarding overall survival were available for 113 patients. Within the follow up time, 89 patients (78.8%) died after a mean follow up time of 22.1 months. Mean follow-up time of patients still alive at the endpoint of analysis was 54.0 months. Cases were staged according to "TNM Classification of Malignant Tumours. 7th edition" [29] and were graded as recommended by the WHO [30].

### Tissue microarray construction

Of all PDACs 3-μm sections were cut and stained with H&E. Three representative areas from the tumor center and invasive margins were marked by a board certified pathologist (W.W.). For each case three tissue cores (1.5-mm diameter) from the selected representative tumor areas were punched out of the sample tissue blocks and embedded into a new paraffin array block using a tissue microarrayer (Beecher Instruments, Woodland, CA).

### Immunohistochemistry

For immunohistochemical detection of Sirt1 on tissue samples, a monoclonal rabbit antibody (dil.: 1:100, clone E104, Cat# 1104–1; Epitomics, Burlingame, CA, USA) was used.

After heat-induced antigen retrieval, slides were incubated with the primary antibody at 4 degree Celsius overnight. Bound antibody was detected by a streptavidin–biotin system (BioGenex, San Ramon, CA, USA). For colour development, a Fast Red system (Sigma, Deisenhofen, Germany) was used. Omission of the primary antibody served as negative control. The slides were cover slipped after counterstaining.

Nuclear staining of Sirt1 was scored by applying a semi-quantitative immunoreactivity scoring (IRS) system, as described previously. Briefly, the intensity of staining and percentage of cells stained were evaluated separately. The IRS for each individual case ranging from 0 to 12 was calculated by multiplication of the intensity and frequency scores. Cases exhibiting an IRS from 0–6 were combined in one group ('Sirt1 low’), cases with an IRS of > 6 were combined in a 'Sirt1 high’ group. Staining of tissue slides was evaluated by experienced pathologists (WW and AS) blinded towards patient characteristics and outcome.

### Cell culture

The human pancreatic cancer cell lines PANC-1 (#CRL-1469) and MiaPaCa-2 (#CRL-1420) were obtained from the American Type Culture Collection (ATCC, Rockville, MD, USA) and cultured in Dulbecco's modified Eagle's medium supplemented with 10% fetal bovine serum and P/S. For the MIA-PaCa-2 cells, additionally 2.5% horse serum and 5 ml NaHCO_3_ (0.75 mg/ml final concentration) were used. These two cell lines were chosen, since PANC-1 is a prototypical Gemcitabine resistant cell line, while Mia-PaCa-2 is known to retain some Gemcitabine sensitivity.

### Reagents

Cambinol (Cat#566323) was purchased from Merck (Darmstadt, Germany), Gefitinib (Cat#PKI-GFTB2-200) was obtained from Biaffin (Kassel, Germany) and Nicotinamide from Sigma (Taufkirchen Germany).

### Plasmids, siRNA and transfections

The SIRT1/2 and GFP control expression constructs were obtained from Addgene. For SIRT1, expression of the FLAG-tagged SIRT1 open reading frame was under the control of an SV40 promotor, allowing physiological levels of SIRT1 expression in cells not harbouring the Large-T antigen (pECE-FLAG-SIRT1, constructed by Michael Greenberg [31]). GFP (Addgene plasmid 13031, constructed by Doug Golenbock) was cloned in a pcDNA3 vector, allowing high protein expression controlled by CMV promotor. Predesigned siRNAs for Sirt1 were purchased from Dhamarcon (ON-TARGETplus SMARTpool, human Sirt1, Cat# L-003540-00-0010). The target sequence is as follows: GCGAUUGGGUACCGAGAUA. A non-target scambled siRNA was used as negative control (all stars negative control siRNA; Cat#1027281, Qiagen, Hilden Germany). After 72 h, the efficacy of transfection was checked by immunoblotting.

All transfections were performed using oligofectamine (dilution: 1:200; Invitrogen, Karlsruhe, Germany) according to the manufacturers’ protocol.

### MTT assay

Cell viability was measured 72 hrs after pSirt1 transfection by the MTT (3-[4,5-dimethylthylthiazol-2-yl]-2,5-diphenyltetrazolium bromide; Sigma, Munich, Germany) assay according to the manufacturer’s instructions. Briefly, 20 μl of 5% MTT solution in PBS was added to each well. After 4-6 h of incubation at 37 °C, the active dehydrogenase in viable mitochondria reduced the tetrazolium ring of MTT to form a blue-colored precipitate, which was then dissolved in 150 μl 50% dimethyl sulfoxide / 50% Ethanol and quantified spectro-photometrically at 570 nm.

### Real time analysis

The PANC-1 and MiaPaCA-2 cell lines were seeded in designated 96 well E-plates (Roche, Penzberg, Germany). Impedance-based real time detection of cellular proliferation was conducted using the xCELLigence system Real-Time Cell Analyzer RTCA-SP (Roche Diagnostics, Penzberg, Germany). The impedance readout as recorded by the xCELLigence system is converted into arbitrary cell index (CI)-values corresponding to each well. The CI value is defined as relative change in measured electrical impedance to represent cell status, and is directly proportional to quantity, size, and attachment forces of the cell. Recording of CI and subsequent normalization of the cell index (normalized cell index, NCI) was performed using the RTCA Software 1.2 (Roche).

The NCI is calculated using the equation: NCI = CI at a given time point divided by the CI at the normalization time point. Hence, the NCI equals 1 at the normalization time point. Background impedance caused by the media was determined in each well before seeding the cells and subtracted automatically by the RTCA software following the equation: CI = (Ri – R0)/15 with Ri as the impedance at any given time point and R0 as the background resistance.

### FACS analysis

The effect of Cambinol and Gefitinib on the cell-cycle profile of pancreatic cancer cells was assessed by flow cytometry. PANC-1 and MiaPaCa-2 were exposed to various concentrations of Cambinol or Gefitinib or combinations thereof for 14 hrs and 72 hrs and the cell cycle profiles were determined by flow cytometry as described previously [32]. Briefly, the cells were harvested with versene, treated with a citric acid buffer (2.1% citric acid and 0.5% Tween 20 in dH_2_O), and stained using a phosphate buffer (pH 8.0, 7.2 g Na2HPO4_ 2H_2_O in 100 mL dH_2_O) containing DAPI. DNA-histograms were obtained by flow cytometry (PAS II, Partec; Muenster, Germany) and the Multicycle program (Phoenix Flow Systems, San Diego, USA) was used for histogram analysis. Each measurement was done in triplicate.

### Immunoblotting

Treated PANC-1 and MiaPaCa-2 cells were lysed in cell lysis buffer (#9803, New England.

Biolabs, Frankfurt, Germany) containing 20 mM Tris–HCl (pH 7.5), 150 mM NaCl, 1 mM Na_2_EDTA, 1 mM EGTA, 1% Triton, 2.5 mM sodium pyrophosphate, 1 mM beta-glycerophosphate, 1 mM Na_3_VO_4_, 1 μg/ml leupeptin as well as Protease inhibitor Mix G (#39101.01; Serva Electrophoresis, Heidelberg, Germany). Prepared protein lysates (30 μg) were denaturated at 95 °C, separated in sodium dodecyl sulfate polyacrylamide (SDS)-polyacrylamide gels (10%) by electrophoresis and electro-transferred to a polyvinylidene difluoride (PVDF) membrane. After transfer, samples were blocked with 5% MP-PBST for 1 h and probed with antibodies against Sirt1 (dil.: 1:5000, clone E104, Cat# 1104–1; Epitomics), cleaved PARP (dil.: 1:300, Cat# 9541, clone Asp214 ; Cell Signaling), pospho-H2AX pSer139 (dil: 1:000, Cat# 05–636, clone JBW 301; Millipore) and beta-Actin (dil: 1:10000, Cat#A5441, clone AC-15; Sigma) diluted in 5 MP-PBST (5% milk powder, Phosphate-buffered saline/Tween) and incubated at 4 °C overnight. The appropriate secondary antibody was applied [1:20000; horseradish peroxidase anti-mouse and horseradish peroxidase anti-rabbit] at room temperature for 1 hr. Visualization was performed by enhanced chemiluminescence (Amersham Bioscience, Freiburg, Germany). Western blots signals were quantified using the ImageJ 1.32 software (National Institutes of Health, Bethesda, MD) after scanning of the films.

### Statistical analysis

For correlation analysis of Sirt1 expression with clinic-pathological parameters, the Fisher’s exact test or χ2 test for trends was applied. For univariate analysis we used the Kaplan-Meier method and a Log-rank test to probe for significance. For multivariate survival analysis the Cox proportional hazard method was used. Variables found in univariate analysis to be significantly related to survival were included in the Cox models. For statistical analysis of cell cycle and MTT data, a two-tailed t-test was applied. For all statistical tests and methods, p-values of <0.05 were considered statistically significant. Statistical analyses were carried out with SPSS 15.0 and Graph Pad Prism 4.

## Results

### Patients’ and tumor characeristics

The patients’ demographics are listed in Table 1. The mean follow-up time was 22.1 months. During the study period, 89 patients died. The median survival was 13.4 months and the median time to death was 10.3 months (range: 1.2 to 41.93 months). 65 patients were below the age of 65 and 64 patients above the age of 65 (median 65 yrs). 118 PDAC were located in the head of the pancreas and 11 in the pancreatic corpus or tail.

**Table 1:** Clinico-pathological characteristics of the PDAC study cohort: correlation with Sirt1 expression

**Characteristics**	**All cases**	**SIRT1 *low***	**SIRT1 *high***	**p-value**
***All cases***				
	129	93 (72.1%)	36 (27.9%)	
***Age***				0.401
≤65 years	65 (50.4%)	48 (73.8%)	17 (26.2%)	
>65 years	64 (49.6%)	48 (70.3%)	17 (29.7%)	
***WHO stage***				0.871
I	12 (9.3%)	9 (75%)	3 (25%)	
IIA	17 (13.2%)	13 (76.5%)	4 (23.5%)	
IIB	91 (70.5%)	66 (72.5%)	25 (27.5%)	
III	5 (3.9%)	3 (60.0%)	2 (40.0%)	
IV	4 (3.1%)	2 (50.0%)	2 (50.0%)	
***Tumour stage***				0.793
T1	/	/	/	
T2	24 (18.6%)	18 (75.0%)	6 (25.0%)	
T3	100 (77.5%)	72 (72.0%)	28 (28.0%)	
T4	5 (3.9%)	3 (60.0%)	2 (40.0%)	
***Nodal status***				0.520
N0	31 (24.0%)	22 (71.0%)	9 (29.0%)	
N1	98 (76.0%)	71 (72.4%)	27 (27.6%)	
***State of metastasis***				0.310
M0	125 (96.9%)	91 (72.8%)	34 (27.2%)	
M1	4 (3.1%)	2 (50.0%)	2 (50.0%)	
***Grade***				0.001
G1	8 (6.2%)	6 (75.0%)	2 (25.0%)	
G2	64 (49.6%)	55 (85.9%)	9 (14.1%)	
G3	57 (44.2%)	32 (56.1%)	25 (43.9%)	

### Sirt1 expression in PDACs

**Figure 1:**
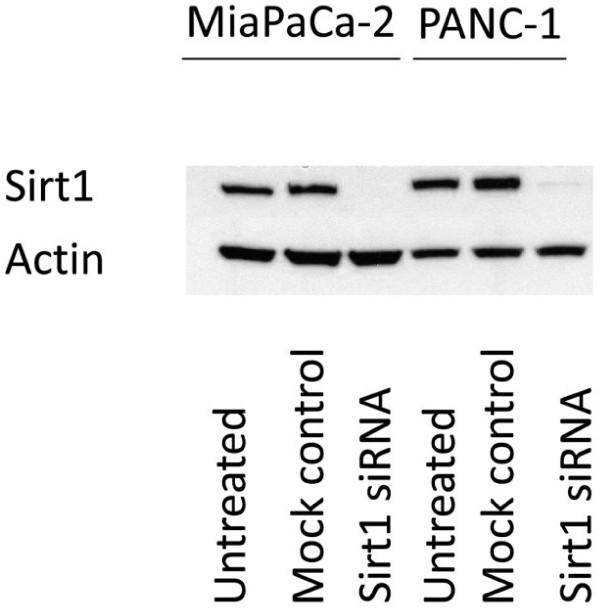
**Immunblots with the antibody against Sirt1.** While endogenous Sirt1 levels were detected by the antibody, knockdown of Sirt1 by siRNA in MiaPaCa-2 and PANC-1 cells led to a complete abrogation of the immunosignal indicating that the antibody binds specifically to its target protein.

The specificity of the antibody used for immunohistochemistry was corroborated by siRNA-mediated knock-down of Sirt1 in MiaPaCa-2 and PANC-1 cells and subsequent immunoblotting with the Sirt1 antibody. The knock-down led to complete abrogation of the immunosignal as shown in Figure 1.

**Figure 2:**
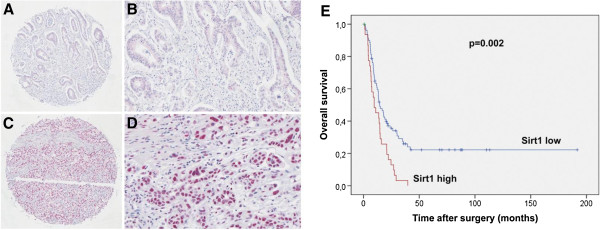
**Sirt1 expression in PDAC. A)** (overview, x5) and **B** (x20) show nuclear Sirt localization with a weak staining signal. **C)** (overview, x5) and **D** (x20) display a strong nuclear Sirt1 immunosignal. **E)** Kaplan-Meier curve for postsurgical survival according to Sirt1 expression. The given p-value was calculated in a log-rank test. The red line indicates tumors of patients with high Sirt1 expression whereas the blue line indicates those with low Sirt1 expression.

As exemplified in Figure 2, we observed a nuclear localization of Sirt1 in PDAC with a low expression (Figures 2A and B) in 72.1% and a high expression (Figures 2C and D) in 27.9% of the cases, respectively. Sirt1 was expressed by tumor cells with varying degrees of nuclear atypia, forming either neoplastic duct like structures, solid masses or single cell infiltrates within desmoplastic stroma.

When analyzed with regard to the morphological features and tumor extent, the expression of Sirt1 was significantly correlated to poor histological differentiation (p = 0.001). There was no statistical difference in Sirt1 expression between early stage and advanced stage tumors (WHO stage and TNM stage, Table 1).

### Univariate survival analysis

By univariate survival analysis (Table 2), patients’ outcome was correlated with both tumor TNM and WHO stage (p = 0.001 and 0.003, respectively). A borderline significance was observed for histological grade (p = 0.058).

**Table 2:** Univariate survival analysis for Sirt1 expression and clinico-pathological parameters in PDAC

		**Cases**	**Events**	**Mean survival (months)**	**Standard error**	**Log-rank-test (p-value)**
***Age at diagnosis***	<=65 years	56	42	50.1	10.5	0.564
	>65 years	57	47	25.1	4.4	
***WHO stage***	I	11	8	29.4	9.8	0.001
	IIA	17	10	52.8	11.9	
	IIB	78	64	33.7	7.0	
	III	3	3	5.6	0.8	
	IV	4	4	11.9	4.1	
***Tumor stage***	T1	/	/	/	/	
	T2	22	17	29.6	6.8	0.003
	T3	88	69	39.6	7.3	
	T4	3	3	5.6	0.8	
***Nodal status***	N0	30	20	43.3	8.8	0.060
	N1	83	69	32.2	6.6	
***State of metastasis***	M0	109	85	42.4	6.8	0.268
	M1	4	4	11.9	4.1	
***Grade***	G1	7	6	16.8	2.5	0.058
	G2	54	39	37.3	6.0	
	G3	52	44	29.7	7.9	
***SIRT1-expression***	low	81	58	54.1	8.9	0.002
	high	32	31	13.0	1.7	

The Kaplan-Meier analysis (Figure 2E) of grouped Sirt1 expression (IRS ≤6, >6) was highly prognostic of poor overall survival for those patients with high Sirt1 expression with a mean postsurgical survival of 13.0 vs. 54.1 months (log-rank test: p = 0.002).

### Multivariate survival analysis

In multivariate Cox regression analysis (Table 3), high Sirt1 expression was significantly related to shorter overall survival (HR 1.647, 95%CI 1.010-2.687, p = 0.045), independently of the degree of histological differentiation and WHO stage.

**Table 3:** Multivariate survival analysis (Cox regression model) including tumor stage and grade in PDAC

	**Overall survival**		
	**HR**	**95%CI**	**p-value**
***WHO stage***			
I	1		
IIA	0.57	0.22-1.45	
IIB	1.11	0.53-2.24	
III	4.92	1.20-20.16	
IV	1.88	0.56-6.33	0.029
***Grade***			
G1/G2	1		
G3	1.28	0.81-2.03	0.298
***SIRT1-expression***			
low	1		
high	1.65	1.01-2.69	0.045

### Cellular effects of Sirt1 overexpression

**Figure 3:**
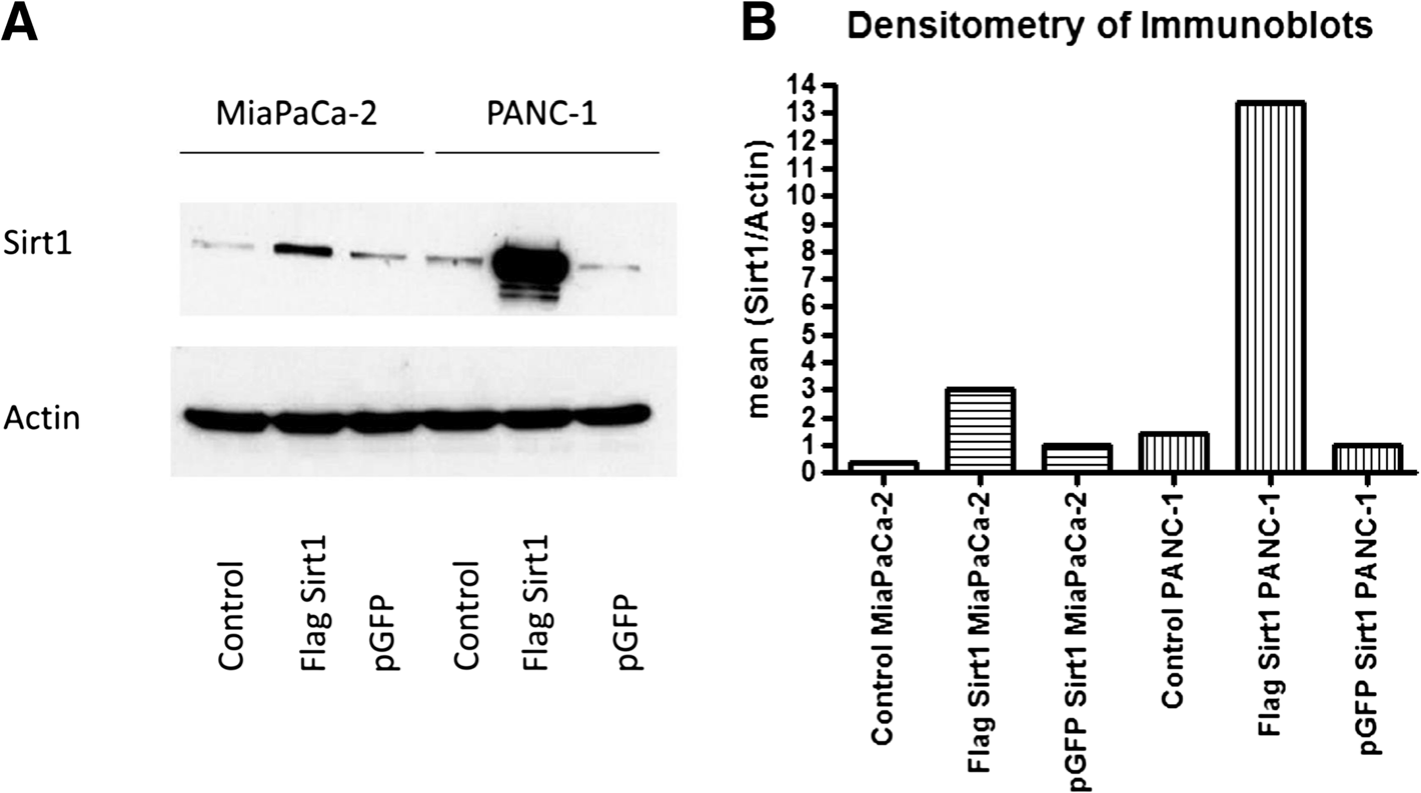
**Immunblots with the antibody against Sirt1 for MiaPaCa-2 and PANC-1. A)** Endogenous protein levels showed a comparably weaker immunosignal than cells overexpressing Sirt1. **B)** The blots were scanned and analysed quantitatively using ImageJ. The values were normalized for pGFP. The graphs show strongly increased immunosignal densities for cells that overexpress Sirt1.

**Figure 4:**
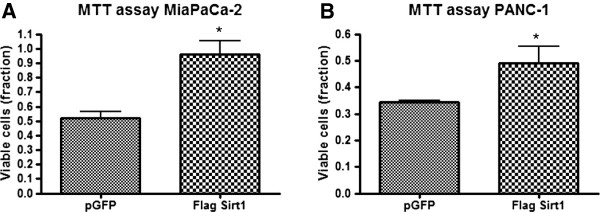
**Cell viability of MiaPaCa-2 and PANC-1 cells assessed by MTT test after treatment with flag-tagged Sirt1 and GFP, respectively. A)** MiaPaCa-2, **B)** Panc-1**.** The test was carried out 3 days after transfection. Bars represent average ± standard deviation (SD) of three independent experiments. *P < 0.05.

To test whether high Sirt1 expression also has a cellular effect *in vitro*, we performed overexpression experiments in both cell lines, MiaPaCa-2 and PANC-1, respectively, using flag-tagged Sirt1. Overexpression of GFP served as control. Figure 3A) shows immunoblots for endogenous and overexpressed Sirt1 in both cell lines. Cells overexpressing Sirt1 showed a markedly stronger immunosignal compared to their untransfected counterparts, which can also be depicted quantitatively as displayed in Figure 3B). Compared to GFP transfected cells, both cell lines showed statistically significantly increased amounts of viable, proliferating cells upon transfection with flag-tagged Sirt1 as determined by MTT assay (Figure 4) and Xcelligence proliferation assays (data not shown).

### Nicotinamide and gefitinib treatment in cells with endogenous or overexpressed Sirt1

**Figure 5:**
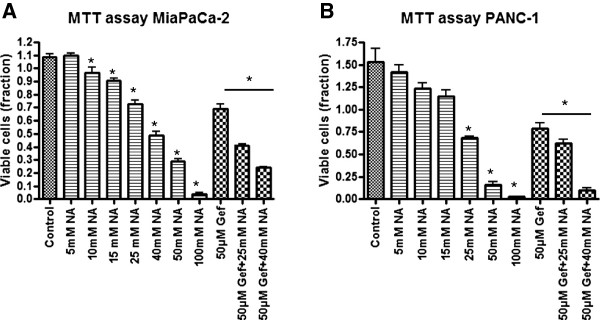
**Cell viability of MiaPaCa-2 and PANC-1 cells assessed by MTT test after treatment with nicotinamide (NA) and gefitinib (Gef), respectively. A)** MiaPaCa2 cells, **B)** PANC-1 cells. Concentrations were used as indicated. Bars represent average ± standard deviation (SD) of three independent experiments. *P < 0.05.

Inhibition of Sirt1 by increasing concentrations of nicotinamide led to a stepwise decrease of viable cells as depicted in Figure 5. Gefitinib treatment with concentrations of 50 μM showed similar effects as observed for the application of 25 mM nicotinamide. Interestingly, combinatory treatment with 50 μM gefitinib and 25 mM or 40 mM nicotinamide showed a synergistic effect on cell viability, which was observed in both cell lines.

**Figure 6:**
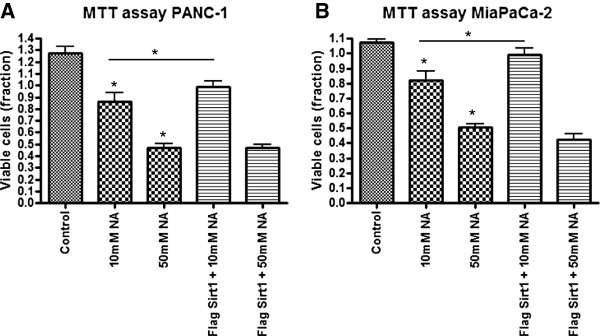
**Cell viability of MiaPaCa-2 and PANC-1 cells assessed by MTT test after treatment with flag-tagged Sirt1 and/or nicotinamide (NA) respectively. A)** PANC-1 cells, **B)** MiaPaCa-2 cells. Concentrations were used as indicated. Bars represent average ± standard deviation (SD) of three independent experiments. *P < 0.05.

Next, we asked whether inhibition of Sirt 1 by nicotinamide may counterbalance the beneficial effect on cell survival triggered by Sirt1 overexpression. We found that application of 10 mM and lower concentrations of nicotinamide, which in untransfected cells already showed a strong decrease of viable cell fractions compared to controls did not influence cell viability in cells overexpressing Sirt1, while higher concentrations of nicotinamide (Figure 6) abrogated increased cell viability mediated by overexpressed Sirt1.

### Cellular effects of cambinol, gemcitabine and gefitinib treatment

#### *Proliferation assay*

**Figure 7:**
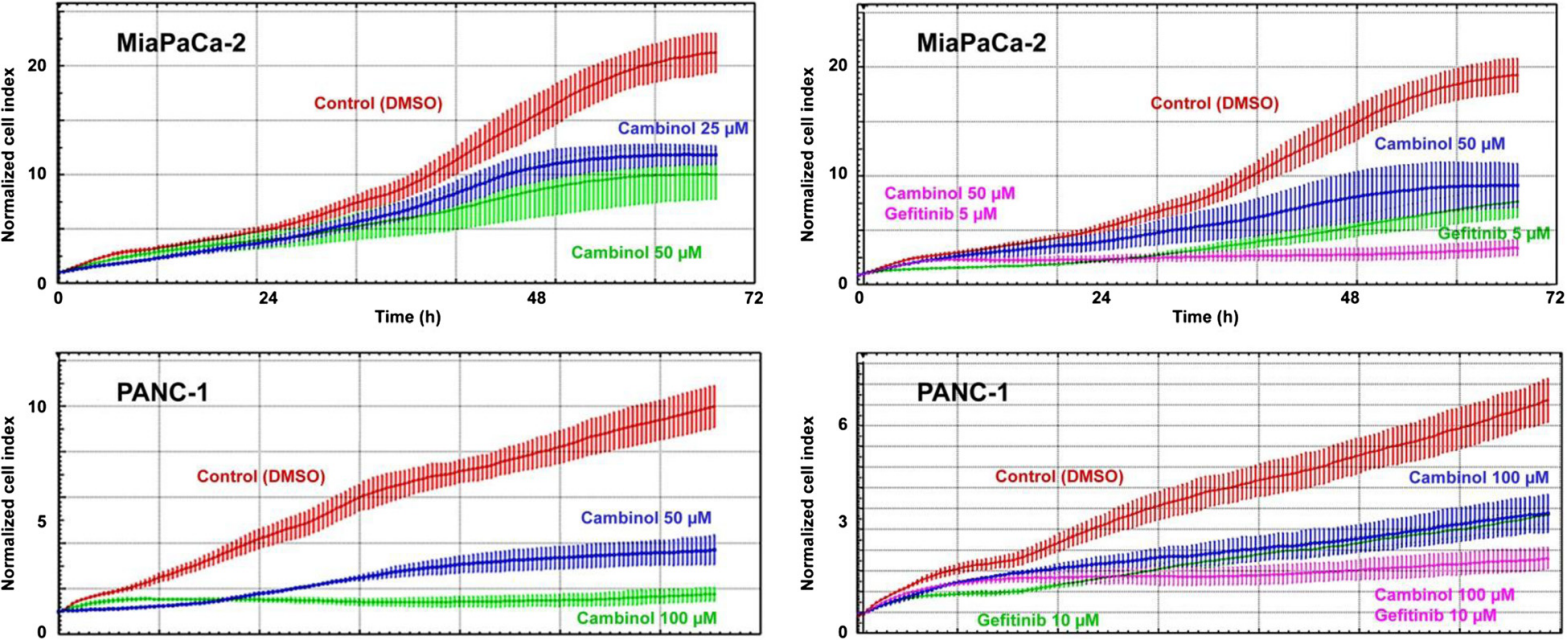
**Real-time cell proliferation assays (X-Celligence system).** Dynamic cell proliferation of MiaPaCa-2 and PANC-1 cells plated on the E-Plates 96 was monitored at 30-min intervals from the time of plating until the end of the experiment.

Real time proliferation assays revealed an inhibition of cell growth of Mia-PaCa-2 cells and PANC-1 cells over a time period of 72 hrs upon treatment with cambinol. While for Mia-PaCa-2 comparably lower concentrations of cambinol (25 and 50 μM) were necessary to achieve this effect, for PANC-1 cells concentrations up to 100 μM had to be applied (Figure 7). Combination of cambinol and gefitinib led to a synergistic inhibitory effect on cell growth for both cell lines. As in the previous experiment slightly higher concentrations for cambinol as well as for gefitinib were used to achieve comparable results in PANC-1 cells.

As expected in Mia-PaCa-2 comparably low concentrations of gemcitabine alone led to strong growth inhibitory effects, while in PANC-1 comparably higher concentrations were necessary (data not shown). Although we tested a multitude of different treatment schemes, a synergistic effect for treatment with gemcitabine and cambinol in combination was not observed (data not shown).

#### *Cell cycle analysis*

**Figure 8:**
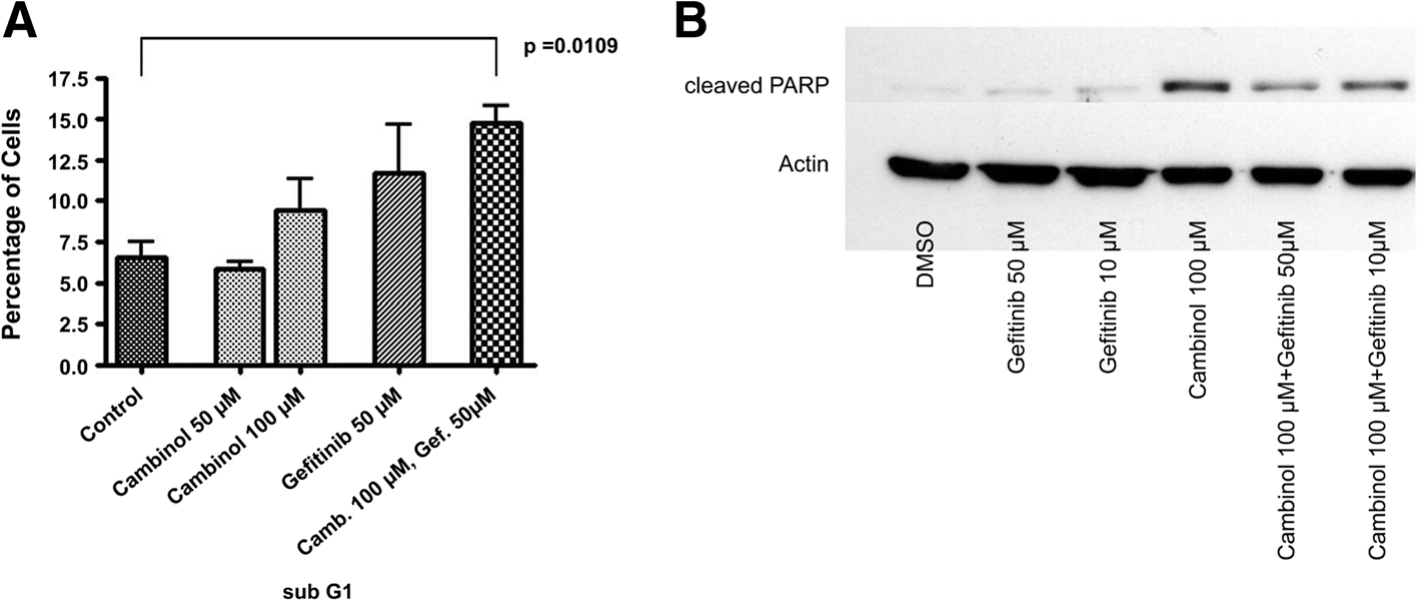
**Cell cycle analysis and apoptosis in PANC-1 cells. A)** Particularly combinatory therapy with gefitinib and cambinol led to a marked sub-G1 peak indicating apoptotic cells. **B)** Immunoblotting for cleaved PARP in PANC-1 cells. Reagents and concentrations as indicated.

To determine the nature of the cellular growth inhibition, we performed FACS analyses. For PANC-1 cells treated with either cambinol or gefitinib alone or in combination, a sub-G1 peak was observed indicating apoptosis (Figure 8A), which was also evident by demonstrating cleaved PARP by immunoblot (Figure 8B). Cell cycle analysis of Mia-Paca-2 cells showed a cell cycle arrest for different concentrations of cambinol (25 and 50 μM) and for a combinatory regimen of cambinol and gefitinib (Additional file 1: Figure S1), but in our experimental setting no apparent apoptosis induction.

#### *Senescence analysis*

Upon treatment with cambinol, we observed for both cell lines a population of growth-arrested cells with a flattened, elongated appearance and extended cellular protrusions (Additional file 2: Figure S2A). As exemplified in Additional file 2: Figure S2B, immunblotting revealed a marked upregulation of y-H2AX in Mia-Paca-2 cells indicating a senescent phenotype.

#### *High concentrations of cambinol lead to abrogation of Sirt1*

**Figure 9:**
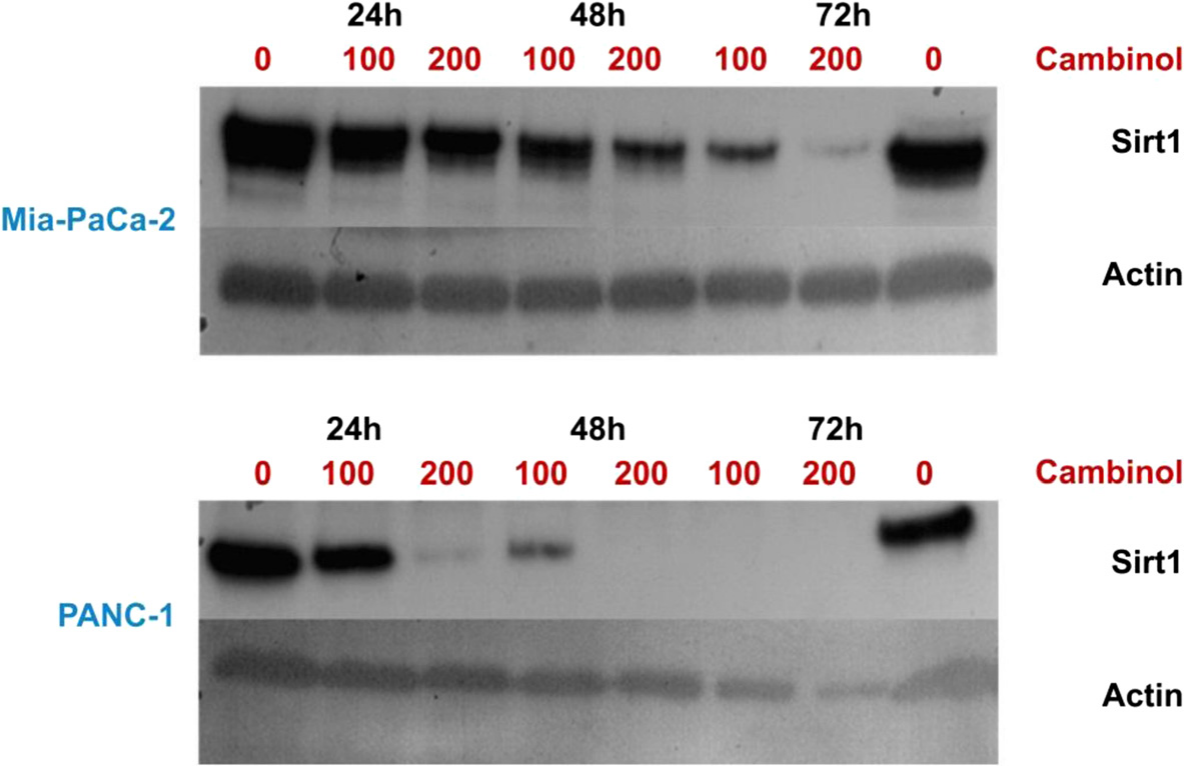
Immunoblots of PANC-1 and MiaPaCa-2 cells show degradation of the target Sirt1 upon cambinol treatment.

Immunoblotting of cells treated with cambinol 100 or 200 μM revealed an extinction of the Sirt1 protein as compared to controls treated with DMSO only (Figure 9). While this effect was repeatedly observed in Mia-Paca-2 cells after 24 hrs, 48 hrs and 72 hrs of cambinol treatment, for PANC-1 cells only high concentrations of cambinol applied for 72 hrs led to a similar effect.

## Discussion

This is the first study that demonstrates Sirt1 to be an independent prognosticator in PDAC with high Sirt1 expression indicating poor outcome. Moreover, our data argue for a functional role of Sirt 1 during tumorigenesis indicating that Sirt1 is not only a biomarker but a potentially oncogenic protein in the PDAC context, whose overexpression leads to increased cell viability in both cell lines, while pharmacological inhibition leads to a concentration-dependent stepwise decrease of viable cells. Cambinol treatment negatively interferes with cell cycle progression (in MiaPaCa-2 cells) and induces apoptosis (in PANC-1 cells) as well as senescence (both cell lines). These observations are in line with Wauters et al. [33] showing an enhancing effect for cell viability and regulatory function of Sirt1 for acinar-to-ductal metaplasia in pancreatic carcinogenesis. The latter results also match data presented by Zhao et al. [28] who reported that utilizing small hairpin RNA Sirt1 knockdown led to increased apoptosis and senescence in PANC-1 cells. However, we failed to observe a synergistic effect of Sirt1 inhibition with Gemcitabine treatment as reported by Zhao et al. [28]. This divergent result may be attributed to the distinct targeting approach in our study, which uses cambinol, a clinically applicable drug with promising anti-cancer effects in animal models of skin cancer and Burkitt’s lymphoma as well as in several cancer cell lines [34]. Interestingly, we detected an application time- and concentration-dependent loss of Sirt1 protein upon cambinol treatment. The underlying cause for this effect, which abrogates Sirt1-function, remains to be elucidated and may be due to protein degradation.

Consistent with the results by Zhao et al. [28] obtained by immunhistochemistry, qPCR and western blotting, we observed a variable expression of Sirt1 in PDACs but did not see a positive correlation of Sirt1 expression with age, tumor size, and lymphatic spread. The different findings may be explained by distinct cohort characteristics including cohort size, age, and sex. However and in contrast to Zhao et al., we observed a strong correlation with higher tumor grades, i.e. the less differentiated the cancer cells are the more Sirt1 expression they exhibit. This finding is of interest since there are reports that implicate Sirt1 in the regulation of cellular differentiation and dedifferentiation processes [35,36]. Dedifferentiation and the associated phenomenon of epithelial-to-mesenchymal-transition play an essential role in the development of early local and distant tumor spread. Observations that link high Sirt1 expression to poorly differentiated cancers were also made by other investigators for hepatocellular carcinoma [37], prostate cancer [38] and glioblastoma [39].

The association between high Sirt1 expression and poor histological grade may also explain why in our cohort Sirt1 expression is associated with poor outcome regardless of the tumor stage as shown by its prognostic independency in multivariate survival analysis. A Sirt1 positive and poorly differentiated tumor may have acquired a biological profile that allows for e.g. early systemic spread of –clinically undetectable- micrometastases in lymph nodes and distant organs leading to impaired survival regardless of the tumor size and metastases detected at the point of initial tumor diagnosis. A recent study by Nalls and colleagues [40] showed that SAHA-induced micro-RNA 34a (miR34a) expression in human pancreatic cancer cells putatively directly inhibited Sirt1 expression by binding within the 3’UTR of Sirt1. On cellular level, restoration of miR34a expression led to growth inhibition as well as decreased epithelial to mesenchymal transition (EMT) and invasion. Although miR34a does not exclusively target Sirt1, this recent study further argues for an oncogenic role of Sirt1 in PDAC development. Recent results obtained by Pramanik et al. corroborate this view [41].

Functional studies indicate that the subcellular localization of Sirt1 might have functional implications in carcinogenesis. Wauters et al. [33] recently provided evidence that there is nuclear to cytoplasmic shuttling of Sirt1 in rat and mouse acinar cells with potential tumorigenic implications in the acinar to ductal metaplasia carcinogenesis model of PDAC. They also reported on cytoplasmic localization of Sirt1 in exocrine cells of the human pancreas. However, investigating human tissue samples of fully developed pancreatic ductal adenocarcinoma, we only detected nuclear localized Sirt1. This may have several reasons. One potential explanation might be that endogenous cytoplasmic Sirt1 levels in comparison to nuclear expression levels are too low to be detected by our antibody. Another explanation would be that cytoplasmic Sirt1 plays a major role in the development of carcinogenic precursors and nuclear Sirt1 has its place in the fully developed cancer. However, this has to be investigated in future functional studies.

Interestingly, following up the seminal work by Luo et al. and Vasiri et al. [6,7], a very recent study by Li and coworkers [42] explored the Sirt1-p53 axis in chronic myeloid leukemia (CML) and found that targeting of Sirt1 by either shRNA or the small molecule inhibitor tenovin-6 resulted in increased levels of acetylated p53 in CML CD34+ cells accompanied by increased transcriptional activity of p53. Abrogation of Sirt1 led to growth inhibition and reduced engraftment of the tumor cells. These effects were even more pronounced when cells were synergistically treated with the tyrosine kinase inhibitor imatinib. These data strengthen the view of a context-dependent tumorigenic impact of Sirt1 as also suggested by our results. Since p53 aberrations are commonly involved in PDAC tumorigenesis [43,44], it is tempting to speculate whether Sirt1 inhibition may help to restore the remaining functionally intact p53 pool. Indeed, recent data [45] indicate that downregulation of Sirt1 by restoration of HIC1 (hypermethylated in cancer 1) leads to increased levels of acetylated p53 and upregulated p21 in pancreatic cancer. On cellular level, overexpressed HIC1, which in turn led to downregulation Sirt1 resulted in cell cycle arrest and apoptosis. Loss of p53 function has also been implicated in resistance to EGFR-targeting strategies [46], the latter having a limited but significant role in the treatment of PDACs [47]. Interestingly, we observed a synergistic impact of combined Sirt1- and EGFR-inhibition suggesting a functional interdependence in PDACs, whose molecular details remain to be explored. In prostatic cancer cells Byles and colleagues [48] observed Sirt1 to modulate EMT upon EGF signalling via the induction of the transcription factor ZEB1. Although it remains to be investigated whether this mechanism works in PDACs, our data and these results may additionally point to a therapeutic rationale for combined EGFR/Sirt1 inhibition.

While a number of small molecule inhibitors of class I and II HDACs are currently in clinical trials for the treatment of malignancies of various organ origins [49], SIRT1 inhibition is currently only investigated in a phase I trial of patients with Huntington’s disease.

## Conclusions

In conclusion, there is accumulating evidence that Sirt1 has an oncogenic role in PDACs and provided that further studies are able to reproduce and extent the data presented herein towards mouse model systems, a clinical trial for patients with PDAC, whose outcome and treatment options are extremely limited for the vast majority of patients, may be worthwhile to consider.

## Competing interest

The authors indicate no potential conflicts of interest.

## Authors’ contributions

AS and WW designed the study, supervised research, analyzed the data, and wrote the paper. AS, VE, and KL performed experiments and analyzed data. V Ehemann performed cell cycle experiments. FK, BS, AW, BG, CK, MB and PN provided patient samples, characterized some of the samples, collected data and assisted in writing the paper. All authors read and approved the final manuscript.

## Pre-publication history

The pre-publication history for this paper can be accessed here:

http://www.biomedcentral.com/1471-2407/13/450/prepub

## Supplementary Material

Additional file 1: Figure S1 Cell cycle analysis of MiaPaCa-2 cells showing growth arrest of tumor cells upon treatment as indicated. Click here for file

Additional file 2: Figure S2 A) PANC-1 and MiaPaCa-2 cells show a flattened phenotype with cellular protrusions. B) Immunoblots of MiaPaCa-2 cells treated with cambinol and gefitinib as indicated showed upregulation of y-H2AX. Click here for file
