# Supplementary figures and images for: High SIRT1 expression is a negative prognosticator in pancreatic ductal adenocarcinoma
Source: BMC Cancer. 2013 Oct 2;13:450. doi: 10.1186/1471-2407-13-450 (PMC3850795; doi:10.1186/1471-2407-13-450)

## Slide 1
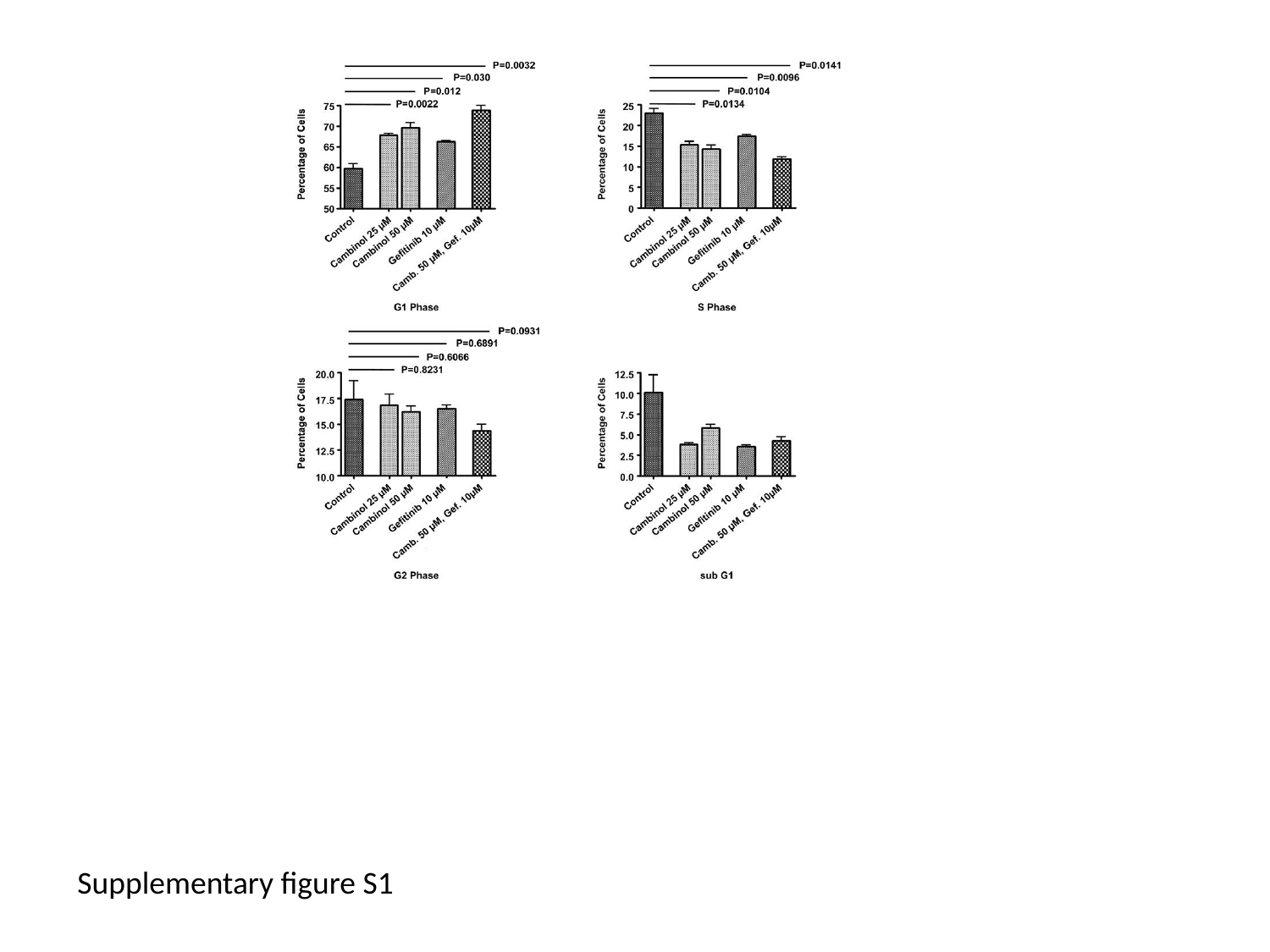

Supplementary figure S1

## Slide 2
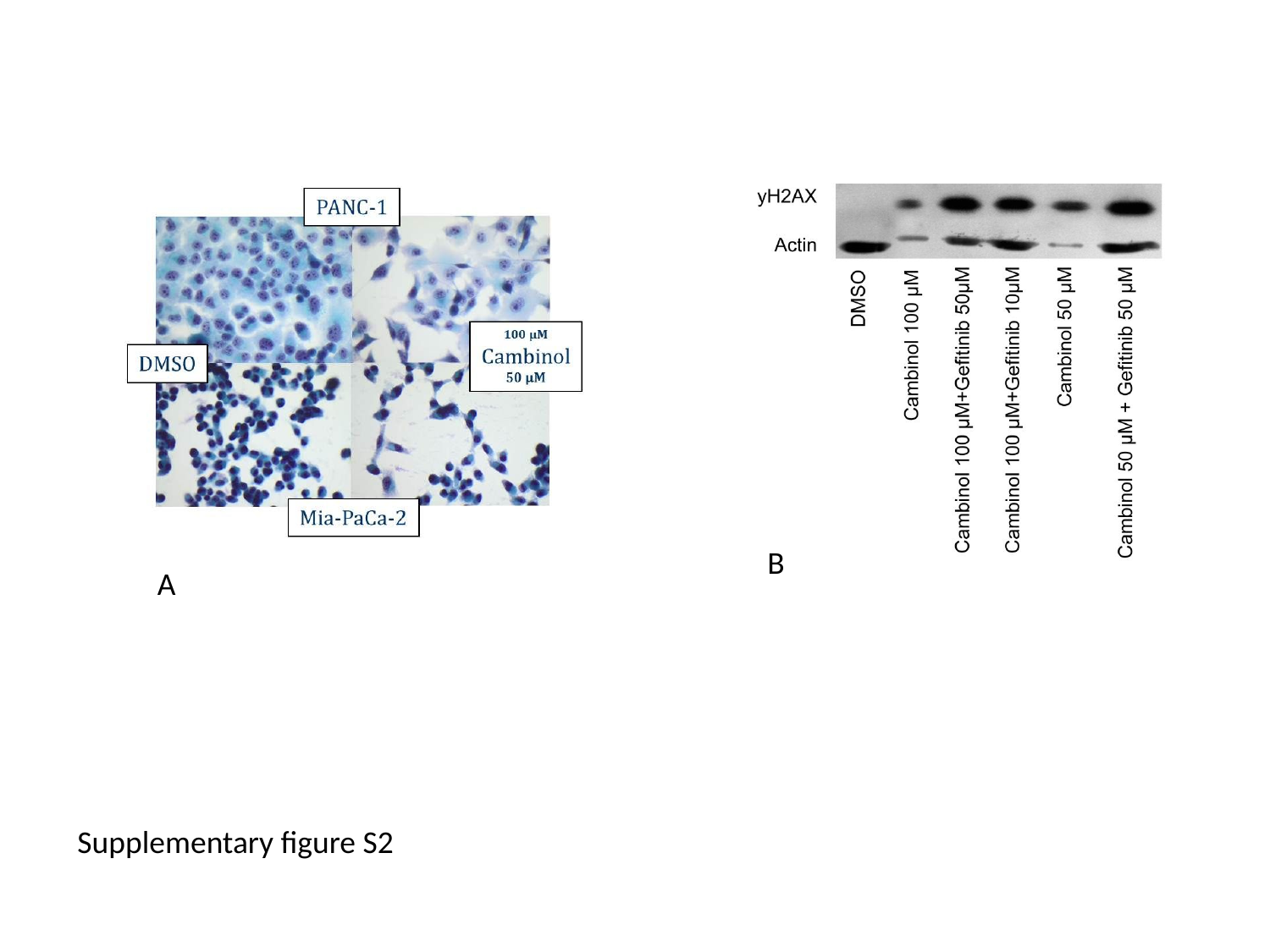

B
A
Supplementary figure S2

Supplement: Additional file 1: Figure S1 — Cell cycle analysis of MiaPaCa-2 cells showing growth arrest of tumor cells upon treatment as indicated. [file 1471-2407-13-450-S1.pptx]

## Slide 1
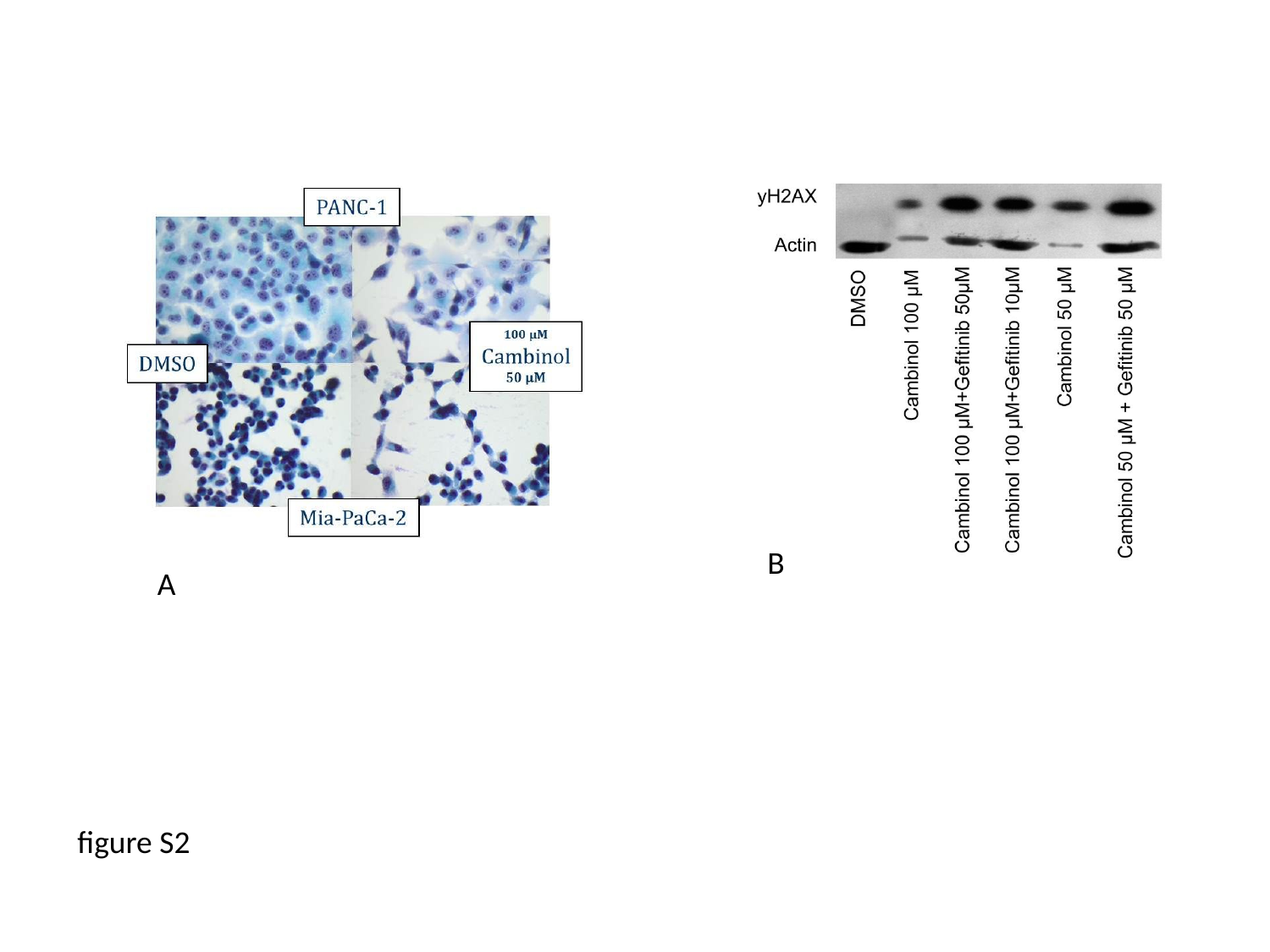

B
A
figure S2

Supplement: Additional file 2: Figure S2 — A) PANC-1 and MiaPaCa-2 cells show a flattened phenotype with cellular protrusions. B) Immunoblots of MiaPaCa-2 cells treated with cambinol and gefitinib as indicated showed upregulation of y-H2AX. [file 1471-2407-13-450-S2.pptx]
